# Management of a Rare Case of Superior Mesenteric Artery Aneurysm Associated with a Pancreatic Cyst Complicated by Acute Rupture: A Case Report and Review of Literature

**DOI:** 10.3390/life14111348

**Published:** 2024-10-22

**Authors:** Petru Razvan Dragulescu, Roxana Carmen Geana (Anghel), Mircea Robu, Reza Nayyerani, Cristian Dumitrescu, Anca Dragan, Catalin Vasilescu, Vlad Anton Iliescu, Ovidiu Stiru

**Affiliations:** 1 Department of Cardiac Surgery, Prof. Dr. C.C. Iliescu Emergency Institute for Cardiovascular Diseases, 022322 Bucharest, Romania; razvan.dragulescu@ymail.com (P.R.D.); reza.nayyerani@drd.umfcd.ro (R.N.); dumitrescu.cristian@aol.com (C.D.); vladanton.iliescu@gmail.com (V.A.I.); ovidiu.stiru@umfcd.ro (O.S.); 2 Faculty of Medicine, Carol Davila University of Medicine and Pharmacy, 050474 Bucharest, Romania; cartalin.vasilescu@umfcd.ro; 3 1st Department of Cardiovascular Anaesthesiology and Intensive Care, Prof. Dr. C.C. Iliescu Emergency Institute for Cardiovascular Diseases, 022328 Bucharest, Romania; anca.dragan1978.14@gmail.com

**Keywords:** superior mesenteric artery, rupture arterial aneurysms, chronic pancreatitis, pancreatic cyst

## Abstract

Superior mesenteric artery aneurysms are a rare pathology, and rupture due to a pancreatic cyst in the context of alcohol-induced pancreatitis is an even rarer condition. The first line of treatment is usually an endovascular approach. We present the case of a 51-year-old male with alcohol-induced pancreatitis, diagnosed with a superior mesenteric artery aneurysm with active bleeding in close contact with a large pancreatic cyst. A covered stent was used to treat this condition. The patient developed hemorrhagic shock 12 h after the procedure and an urgent laparotomy was performed. A second rupture of the arterial wall at the distal end of the stent was observed and in order to obtain distal perfusion, first, an infrarenal aorta to superior mesenteric artery bypass distal to the rupture was performed in order to exclude the aneurysm. Secondly, a bypass originating from the distal end of the first bypass to the distal end of the superior mesenteric artery was performed. The patient had an uneventful recovery and was discharged after 10 days. We reviewed the literature regarding the incidence and the therapeutic management of superior mesenteric artery aneurysm complicated by pancreatic cyst. An advanced search on PubMed from 2004 to 2024 returned 194 results and after applying the inclusion–exclusion criteria, 11 publications were selected. Although the endovascular approach is usually the first line of treatment with obvious advantages for the patient, a patient-tailored approach should be made in such cases and surgery could be the first option, when considering that the mechanism of aneurysm rupture is due to erosion of the arterial wall by the pancreatic enzymes. Surgery has the advantage of cyst drainage and aneurysm exclusion and in our case proved lifesaving.

## 1. Introduction

Superior mesenteric artery (SMA) pseudoaneurysms, though rare, present significant clinical challenges due to their potential for life-threatening complications such as rupture and hemorrhage [1,2,3,4]. These pseudoaneurysms are often associated with underlying conditions, particularly pancreatitis, which can erode the arterial wall through the release of pancreatic enzymes. Understanding the pathophysiology of SMA pseudoaneurysms is crucial, as timely diagnosis and intervention can markedly improve patient outcomes [4,5,6,7,8].

Current diagnostic techniques, especially advanced imaging modalities like computed tomography (CT) and CT angiography, have revolutionized the management of SMA pseudoaneurysms. These methods allow for precise visualization of vascular anatomy and the identification of aneurysms, facilitating early intervention. In particular, CT angiography offers a non-invasive means to evaluate the presence and extent of aneurysms, guiding the choice between endovascular and surgical treatment strategies [9,10,11].

From a therapeutic perspective, endovascular approaches have gained prominence as first-line treatments due to their minimally invasive nature and reduced recovery times. Techniques such as covered stent placement allow for immediate exclusion of the aneurysm while preserving blood flow to the distal vascular bed. However, the anatomical complexity often seen in patients with pancreatic pathology can complicate these interventions, necessitating a thorough understanding of the vascular anatomy and the potential for anatomical variations [9].

Despite advancements in treatment modalities, the management of SMA pseudoaneurysms remains challenging. The risk of rupture, especially in the context of pancreatitis, underscores the importance of personalized medicine approaches tailored to individual patient circumstances. This includes considering the patient’s overall health, the extent of vascular involvement, and the presence of comorbid conditions [10,11].

Therefore, we performed a literature review regarding the incidence of SMA aneurysm in association with pancreatic cyst and its therapeutic management. This review aims to comprehensively explore the current state of knowledge regarding SMA pseudoaneurysms, emphasizing the interplay between advanced imaging techniques and therapeutic strategies, while highlighting the critical role of personalized medicine in optimizing patient outcomes.

## 2. Case Report

A 51-year-old male patient was admitted to the emergency department for severe epigastric pain, nausea, and vomiting with a 2-day onset. His medical history was consistent with alcohol-induced chronic pancreatitis with several episodes of acute decompensation. Physical examination revealed diffuse abdominal tenderness with slight muscular defense. On palpation, an epigastric mass was objectified, and abdominal auscultation revealed normoactive intestinal sounds. The patient denied any transit modifications, fever, or shivers. On admission, the patient had a BMI of 22.5 kg/m^2^ and was hemodynamically stable with a blood pressure of 140/65 mmHg, heart rate of 83 bpm, normal sinus rhythm, and peripheral oxygen saturation of 97% in atmospheric air. Laboratory blood tests revealed elevated pancreatic enzymes: Amylase 91 U/L, Lipase 32 U/L (10 days prior to admission, they were 288 and 147 on a routine check-up). Also, the patient was anemic (Hb 9.5 g/dL), had a GGT of 134 U/L, and had an inflammatory syndrome (fibrinogen 465 mg/dL, and C Reactive Protein 102 mg/L).

**Figure 1:**
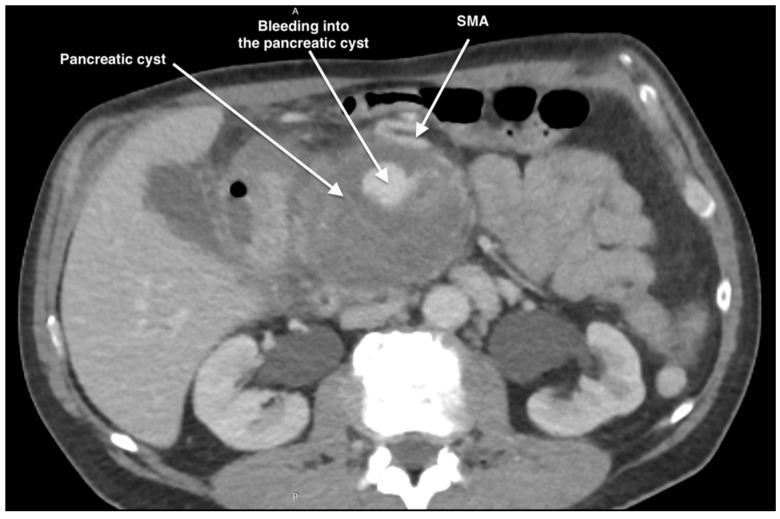
Preoperative CTA aspect of the ruptured SMAA-coronal view describing the course of the SMA and its rupture into the pancreatic cyst.

**Figure 2:**
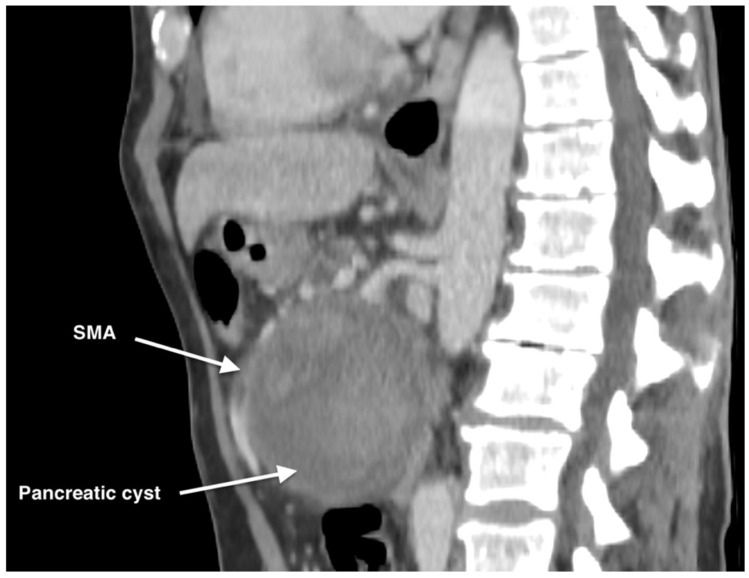
Preoperative CTA aspect of the ruptured SMAA and the pancreatic cyst-sagittal view describing the course of the SMA along the pancreatic cyst.

The computer tomography (CT) scan revealed an SMA aneurysm with a small bleeding observed into a large size pancreatic cyst of 92 mm × 110 mm in the cephalic region (Figure 1 and Figure 2). Another pancreatic cyst of 70 mm × 90 mm in the caudal region and also a right accessory hepatic artery (RAHA) proximally to the aneurysm were found.

**Figure 3:**
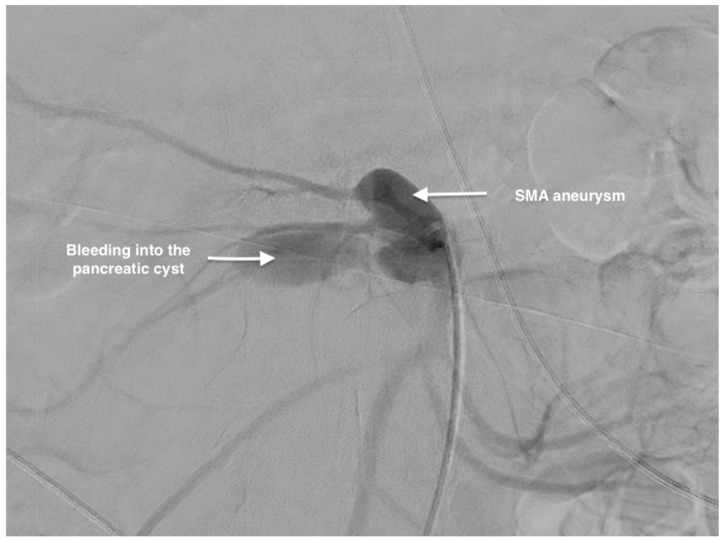
Preoperative arteriography of the superior mesenteric artery and its branches showing the bleeding of the SMAA into the pancreatic cyst.

**Figure 4:**
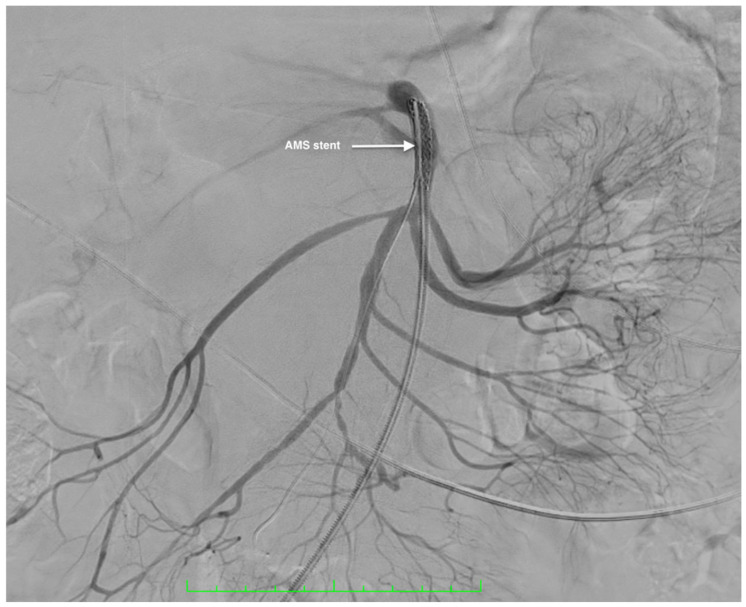
Preoperative arteriography aspect of the SMA after the endovascular treatment of the SMAA showing the Ivascular double-covered stent’s position in the superior mesenteric artery.

Angiography was performed (Figure 3) and the decision to implant a double-covered stent (Ivascular 5 × 37 mm) in the SMA was made. The endovascular approach was without any periprocedural complications and with a favorable result (Figure 4).

After twelve hours from the endovascular procedure, the patient became severely hypotensive, with a blood pressure of 78/60 mmHg. Blood tests revealed a hemoglobin level of 4 g/dL and the patient was immediately transferred to the operating room for urgent exploratory laparotomy.

### Surgical Technique

**Figure 5:**
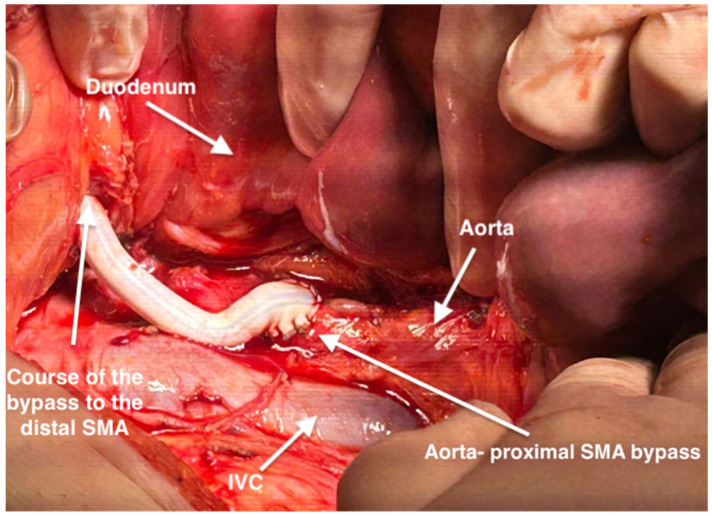
Medial visceral rotation in order to expose retroperitoneal structures. Intraoperative view of the bypass from the aorta to the proximal portion of the superior mesenteric artery. IVC-inferior vena cava.

**Figure 6:**
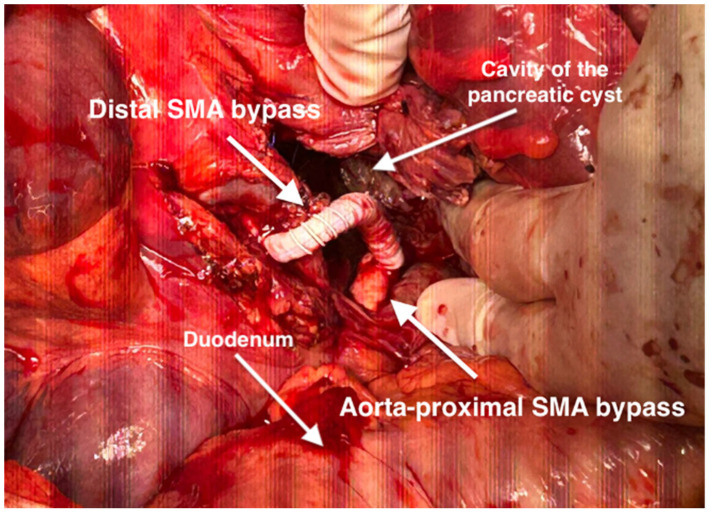
Intraoperative aspect of the bypass from the proximal segment of the SMA to the distal segment of the SMA. The cavity of the drained pancreatic cyst is visible.

After rapid induction of general anesthesia, a median laparotomy was done. We suspected that the cause of the hemorrhagic shock was the rupture of the SMA aneurism. In order to access the origin of the SMA, the Cattell-Braasch maneuver was performed for right-sided medial visceral rotation. The retroperitoneum was incised and the SMA course was identified. A pancreatic cyst was found in direct contact with the wall of the SMA aneurysm. Proximal and distal isolation of the SMA was performed with vessel loops. The splenic artery with the origin from SMA was isolated and clamped. Prior to arterial clamping, 100 IU per kilogram of unfractionated heparin was administered. After clamping of the SMA near the aorta, the cyst was incised and a mix of old and fresh blood was found. Visceral protection was achieved via the delivery of 1000 mL of cold (4C) Custodiol solution into the distal SMA, using a 14Fr Foley catheter. The stent was identified and the initial arterial wall rupture was also identified at the medial portion of the stent. At the distal end of the stent, we identified a second rupture in a friable area of the wall. This second rupture was not seen in the initial angiography study and we presumed that it was caused by the continuing erosion of the pancreatic enzymes. After stent removal and aneurysm exclusion by proximal and distal ligation, an end-to-side anastomosis with a 6 mm ePTFE graft with a 6.0 Polypropylene suture was performed distal to the ruptured artery wall. The proximal end of the graft was implanted with the same technique in the infrarenal aorta. Intraoperative epivascular Doppler of the distal SMA revealed a low distal flow with good perfusion of a hepatic accessory artery that originated from the SMA aneurysm. In order to address this distal visceral hypoperfusion, another interposition with a 6 mm ePTFE graft was performed from the distal end of the original bypass to the distal end of the SMA. Epivascular Doppler ultrasound of the distal SMA was within normal limits (Figure 5 and Figure 6). The pancreatic cyst was drained and a drain tube was placed in the proximal area. The surgery was concluded in the usual manner.

The patient was transferred to the intensive care unit (ICU) and had a favorable postoperative evolution. He was extubated at 8 h postoperatively and was hemodynamically stable without the need for vasopressors. Intravenous anticoagulation started 3 h from surgery. Serum lactate and pancreatic enzymes were checked every 3 h in the first 24 h, with no detected elevation. Maximum lipase level was 30 U/L and maximum amylase level was 40 U/L. Serum lactate had a peak of 2.2 mmol/L. Oral anticoagulation intake was started on postoperative day four with a target INR between two and three. On the eighth postoperative day, the drains were removed at a drainage of 50 mL/24 h and after normal intestinal transit.

**Figure 7:**
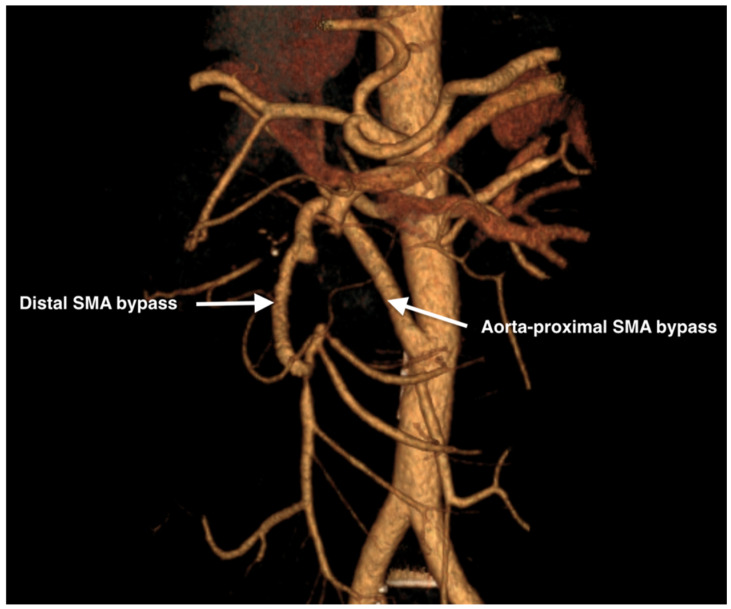
Postoperative 3D reconstruction of the computed angiography of the aorta and the SMA showing the patency of the bypass.

Postoperatively contrast enhanced CT evaluation showed patent SMA bypasses with good distal perfusion of the SMA and its branches (Figure 7). The patient was discharged 10 days postoperatively and remains asymptomatic.

## 3. Discussion

Visceral artery aneurysm is a rare condition. Despite a low incidence, it is associated with a high mortality rate upon rupture, estimated at 25–75% [1,5]. The incidence of this condition rose in recent years mostly due to imaging techniques and the aging population [3,4,11]. Regarding SMA aneurysms, the prevalence is unclear and it is estimated between 0.5 and 2 per 10,000 [12,13].

The definition of visceral aneurysms includes both true aneurysms and pseudoaneurysms, but their etiologies and natural evolution are very different. It appears that SMA aneurysms tend to affect predominantly men, with the average age of presentation of 50 years [5,11,14,15]. Infection is the most common etiology of SMAAs. Mycotic aneurysms sum up for almost 60%. Pseudoaneurysms of the SMA are rare, trauma, or inflammation in the context of pancreatitis being the most frequent cases [14,16,17].

Superior mesenteric artery pseudoaneurysm in patients with pancreatitis is a rare vascular complication of pancreatitis. Various mechanisms have been proposed to explain the formation of pseudoaneurysms, with the two most widely accepted theories being: the presence of a pseudocyst that erodes and weakens the wall of a nearby artery, leading to the development of a pseudoaneurysm, and the activation of proteolytic enzymes within the pancreatic tissue, which results in the auto-digestion of pancreatic and adjacent structures. When a pseudoaneurysm ruptures, it results in uncontrolled hemorrhage into the abdominal cavity, which can rapidly lead to hypovolemic shock and death if not promptly managed. The risk of rupture is heightened by the ongoing enzymatic activity and the inflammatory environment created by pancreatitis [8,15,18,19].

Other possible etiologies of SMA aneurysms are connective tissue diseases such as the Marfan syndrome. Patients with the Marfan syndrome usually present with aortic root dilatation associated with aortic insufficiency, but the aneurysmal development may occur at any level of the aorta including the SMA. These patients usually have a multi-stage treatment approach because the pathological alterations of the arterial wall continue to develop. Because of this fact, subsequent surgeries at other sites besides the thoracic aorta are needed for a significant number of patients [20].

In our case, the etiology of the SMA pseudoaneurysm is most likely due to alcohol-induced pancreatitis considering the patient’s past medical history, with multiple decompensation episodes. The mechanism implicated in the rupture of the SMA pseudoaneurysm that led to the hemorrhagic shock could be the weakening of the arterial wall by the pancreatic enzymes released from the level of the cyst. Also, mechanical stress could contribute to rupture of the SMA aneurysm when considering the close proximity of the cyst to the wall of the SMA.

The clinical presentation of a ruptured SMA pseudoaneurysm can be dramatic and nonspecific, often complicating early diagnosis. Patients typically present with sudden, severe abdominal pain, which may radiate to the back. Symptoms of hemorrhagic shock are common. Additionally, patients may exhibit signs of gastrointestinal bleeding, such as hematemesis or melena, if the rupture communicates with the gastrointestinal tract.

Given the overlap in symptoms with other abdominal emergencies, a high index of suspicion is necessary, especially in patients with a history of pancreatitis or known pancreatic pseudocysts.

One of the major differential diagnoses that must be taken into consideration are the abdominal complications of acute aortic dissection, such as bowel malperfusion caused by superior mesenteric artery dissection. In such cases, the patient’s treatment approach must be individualized based on multiple considerations like the entire aortic anatomy and the static or dynamic nature of the intimal flap obstruction, and it may consist of classic surgical treatment combined with percutaneous endovascular techniques in order to achieve a full blood flow restoration [21].

Accurate and rapid diagnosis of a ruptured SMA pseudoaneurysm is crucial for effective management with imaging studies playing a main role in this regard.

Computed tomography (CT) or computed tomography angiography (CTA) is recommended as the first-line choice for diagnosis and treatment planning due to its advantages in depicting the relationship between aneurysms and associated blood vessels, enabling 3D vascular reconstruction, and supplying essential data for determining the appropriate surgical approach. Computed tomography angiography is also considered essential in evaluating the entire aorta in order to diagnose the special cases of superior mesenteric artery aneurisms that occur in connective tissue disorders such as the Marfan syndrome.

### Review of Literature

We conducted a review of the literature on PubMed database from 2004 to 2024. The main objective of the research was to identify the incidence and therapeutic management of superior mesenteric artery aneursysm associated with pancreatic cyst. We conducted an advanced search on PubMed using the syntax: (superior mesenteric artery aneurysm) AND ((pancreatic cyst) OR (pseudopancreactic cyst) OR (pancreatic cyst rupture) OR (pancreatitis)), which returned 194 results, and after applying the inclusion and exclusion criteria, we selected 11 articles. The inclusion criteria consisted of the following: relevant manuscript for the search topic; full manuscript published in recognized journals; and manuscripts that included the management of this association of these pathologies. The exclusion criteria were represented by the following: publications that were not relevant to the topic; and letters, covers, correction or comments to published manuscripts.

The reported rupture rate for superior mesenteric artery aneurysms is approximately 38%. However, the exact rupture rates for less common superior mesenteric artery aneurysms remain unclear [22].

Visceral artery pseudoaneurysms always require treatment, regardless of their size or symptoms. Table 1 highlights the main studies dealing with the management of SMA aneurysms. Conservative management is not advised due to the high rupture rate and up to 90% mortality in untreated cases. The primary goal of treatment is to exclude the aneurysm sac. As stated in the Society of Vascular Surgery guidelines, once diagnosed, a SMAA should be treated as soon as possible [23].

The main treatments for superior mesenteric artery aneurysm include open surgery, endovascular surgery, and conservative management.

Classical open surgical treatments include simple aneurysm resection or resection combined with revascularization. The choice of surgical method depends on the aneurysm’s location, whether it is ruptured, and its relationship with the branching vessels. The challenges of open surgery lie in the complex anatomy of the superior mesenteric artery aneurysm and the difficulty of achieving a good surgical field.

With advances in interventional techniques, endovascular surgery has become a common approach for treating superior mesenteric artery aneurysms in recent years. This includes procedures such as covered stent grafts, multi-layer bare stent implantation, and coil or embolic material embolization. However, the limitations of endovascular treatment originate primarily from the complex anatomy of the superior mesenteric artery, which leads to an increased risk of covering critical branches at the main trunk of the superior mesenteric artery and also poses a real challenge in maneuvering the guide wire and catheter to the lesion site on distal branches [24,25].

The endovascular treatment is deemed safer, especially in patients with multiple comorbidities, but it should not be used when an infectious etiology is suspected. The location of the SMA aneurysm is of the utmost importance, because it may be difficult to place a stent near the origin owing to the lack of an adequate landing zone. Large aneurysms may not be suitable to the use of a covered stent owing to instability in a large lumen. The stent should extend at least 1 cm proximally and distally to achieve a complete seal.

Coil embolization is also an option to treat SMAA, but it carries the risk of incomplete coiling, leading to aneurysm progression. Bare stent-assisted coiling is deemed safer and does not bear the risk of side branch obstruction. Stent-assisted coiling requires longer procedure times and higher doses of radiation. Covered stent implantation is safe when anatomy is favorable, and there is no risk of side branch occlusion. All the necessary measurements can be determined from the preoperative CT angiogram and should be compared with the diagnostic angiogram obtained before the endovascular procedure [22,26]. Table 1 summarizes the main studies regarding the management of SMA aneurysms.

From the selected studies, 45.4% [5] were published in the last five years, showing that the incidence of the association of these pathologies is increasing. This may be due to the development of imagistic evaluation, which leads to an earlier and more accurate diagnosis.

Regarding the management of the patients included in the selected studies, 36.3% [4] of the publications described the surgical approach, 36.3% [4] used endovascular interventions, and 27.2% [3] combined the surgical and endovascular treatments. The studies that used endovascular procedures, alone or combined with surgery, were more recently published. The treatment strategy in this association of pathologies is guided by the patient’s status. In our case, the patient became hemodynamically unstable; he was in a hemorrhaging shock, with hemoglobin levels of 4 g/dL, and we decided on an emergent surgical approach. In some situations, the endovascular treatment can stabilize the hemodynamic status of the patient. In those cases, endoscopic transpapillary drainage or endoscopic ultrasound drainage can be considered as a treatment option for the pancreatic pseudocyst.

The management of a ruptured SMA pseudoaneurysm requires a multidisciplinary approach and often involves both immediate resuscitative measures and definitive surgical or endovascular intervention. In our case, we initially opted for an interventional approach with a covered stent, considering that the patient was stable and only a small quantity of contrast agent was observed extravasated from the aneurysm. However, this strategy proved to be insufficient with the patient entering into hypovolemic shock, suggesting that a classical exploratory laparotomy is needed. Intraoperatively, we have found a second rupture at the distal end of the stent, which was not initially seen in preoperative studies, demonstrating the rapidly evolutive nature of this condition. If the initial rupture was with high probability caused by the erosion of the pancreatic enzymes from the cyst into the wall of the SAM, the question arises as to whether the placement of the stent could have contributed to the further weakening of the SMA wall. The location of the second rupture, at the distal end of the stent, could suggest this. Considering this, we could say that surgical management from the beginning could have been a more suitable approach when dealing with a ruptured SMAA caused by a pancreatic cyst.

In our case, the etiology of the SMA pseudoaneurysm was likely alcohol-induced pancreatitis, resulting in recurrent episodes and eventual formation of the aneurysm. The clinical presentation can be ambiguous, with symptoms of severe abdominal pain and signs of hemorrhagic shock complicating diagnosis. A high index of suspicion is vital, particularly in patients with known pancreatitis or pancreatic cysts.

This case highlights the need for personalized treatment strategies. While the initial approach in our patient involved endovascular stenting, the subsequent rapid deterioration necessitated surgical intervention, emphasizing the dynamic nature of SMA pseudoaneurysms. Given the potential for complications, a surgical approach may sometimes be more appropriate from the outset, particularly in cases with significant anatomical concerns.

In the presented case, both major indications for open surgical treatment were met, firstly because the patient was hemodynamically unstable, in hypovolemic shock, and secondly because our patient had previously received endovascular treatment for the SMAA.

The prognosis for patients with a ruptured SMA pseudoaneurysm is uncertain, with outcomes heavily dependent on the timing of diagnosis and intervention.

## 4. Conclusions

A ruptured superior mesenteric artery pseudoaneurysm associated with pancreatitis represents a rare and life-threatening medical and also surgical emergency. Early recognition, prompt imaging, and fast intervention are critical in order to improve patient outcomes. A patient-tailored management should be considered and in the particular case of an SMA aneurysm in close contact with a pancreatic cyst, a surgical approach as the initial treatment could be a safer option.

From the review of the literature, we concluded that advances in imaging and endovascular techniques have enhanced the diagnostic and therapeutic capabilities for this condition, but the high morbidity and mortality rates underline the need for ongoing active follow-up and multidisciplinary collaboration in managing complex pancreatic and vascular pathologies. Further studies with long-term follow-up are necessary in order to evaluate the best therapeutic strategies.

## Figures and Tables

**Table 1:** Main studies regarding the management of SMA aneurysm. SMAA: superior mesenteric artery aneurysm; NS: not specified; NA: not available.

Authors	Year	Country	Number of Patients	Mean Age (Years)	SMAA	Pancreatic Cyst	Treatment	Follow-Up (Months)	Mortality
Pulli et al. [12]	2008	Italy	55	59.3	2	1	Surgery	120	50%
Wang et al. [17]	2020	China	18	49.1	18	2	Surgery 9 pts Endovascular 3 pts Conservative 3 pts	47	0%
Zyromski et al. [16]	2007	USA	35	59.3	3	3	Surgery	NS	NS
Guo et al. [24]	2016	China	8	55.3	8	1	Endovascular	NS	NS
Arias et al. [7]	2020	Spain	1	NS	1	1	Endovascular	NS	NS
Saftoiu et al. [11]	2004	Romania	1	66	1	1	Surgery	NA	100%
Bouassida et al. [18]	2012	Tunisia	1	50	0	1	Surgery	NA	100%
Kaszczewski et al. [2]	2023	Poland	1	41	1	0	Endovascular	12	0%
Pitcher et al. [4]	2022	USA	131	60	41	1	Surgery 36 Endovascular 8	43.6	NS
Okubo et al. [10]	2023	Japan	1	92	1	0	Endovascular	21	0%
Saltzberg et al. [13]	2005	USA	65	64.9	3	0	Endovascular 18 (27.7%) Surgery 9 (13.9%)	14.9	NS

## Data Availability

Data available on request.
